# Asymptotic Analysis of SPTA-Based Algorithms for No-Wait Flow Shop Scheduling Problem with Release Dates

**DOI:** 10.1155/2014/979238

**Published:** 2014-02-09

**Authors:** Tao Ren, Chuan Zhang, Lin Lin, Meiting Guo, Xionghang Xie

**Affiliations:** ^1^Software College, Northeastern University, Shenyang 110819, China; ^2^School of Business Administration, Northeastern University, Shenyang 110819, China

## Abstract

We address the scheduling problem for a no-wait flow shop to optimize total completion time with release dates. With the tool of asymptotic analysis, we prove that the objective values of two SPTA-based algorithms converge to the optimal value for sufficiently large-sized problems. To further enhance the performance of the SPTA-based algorithms, an improvement scheme based on local search is provided for moderate scale problems. New lower bound is presented for evaluating the asymptotic optimality of the algorithms. Numerical simulations demonstrate the effectiveness of the proposed algorithms.

## 1. Introduction

The no-wait flow shop is a very typical scheduling setting in steel production, food production, service industry, and so forth [1]. In this scheduling problem, *n*  (*n* ≥ 1) jobs have to be processed on a set of *m*  (*m* ≥ 2) machines following the same route. There is no intermediate storage between any two adjacent machines. The finished job has to remain on the upstream machine, until the downstream machine becomes available. The jobs to be processed arrive to the system over time. The goal is to achieve a job sequence that minimizes the sum of total completion times. For formally stating the scheduling problems, the standard three-field notation [2] is employed in the succeeding content.

Röck [3] reported the strong NP-hardness for problem F2*|*no-wait*|*Σ*C*_*j*_. As the F2*|*no-wait*|*Σ*C*_*j*_ problem is a special case of the F*m|*no-wait, *r*_*j*_ *|*Σ*C*_*j*_ problem, it implies that obtaining the optimal solution in polynomial time for the latter is impossible. Wang et al. [4] considered the F*m|*no-wait*|*Σ*w*_*j*_(1 − *e* ^−*rC*_*j*_^) problem, where *w*_*j*_ is the weight of job *j* and *r* is the discounted rate. The authors developed efficient polynomial time algorithms for finding the optimal schedules of the problem. Su and Lee [5] investigated a no-wait and separate setup two-machine flow shop system with a single server (i.e., F2 S1*|*no-wait, *s*_*j*_ *|*Σ*C*_*j*_). They proposed optimal solutions for several restricted cases and some properties for general case as well as established the heuristic and branch and bound algorithms to tackle the problem. Huang et al. [6] addressed a no-wait flow shop problem with two-stage multiprocessor (i.e., F2(P1, P2)*|*no-wait, *s*_*j*_ *|*Σ*C*_*j*_, where *s*_*j*_ is the setup time of job *j*). They implement integer programming model and an ant colony optimization to test, analyze, and compare simulated data. Framinan et al. [7] proposed a new constructive heuristic based on an analogy with the two-machine problem in order to select the candidate to be appended in the partial schedule. They conducted computational tests to show that the proposed heuristic outperforms existing ones. Zhao and Tang [8] extend the artificial immune system approach by proposing a new methodology termed as Psycho-Clonal algorithm to deal with problem F*m|*no-wait *|*Σ*C*_*j*_. Qian et al. [9] presented a hybrid algorithm based on differential evolution (HDE) to solve the F*m|*no-wait, *s*_*jk*_, *r*_*j*_ *|*Σ*C*_*j*_, where *s*_*jk*_ denotes the sequence dependent setup time. The superiority of HDE in terms of searching quality, robustness, and efficiency is demonstrated by simulations.

For the unlimited buffer version of this problem (i.e., F*m|* *r*_*j*_ *|*Σ*C*_*j*_) Liu et al. [10] reported the asymptotic optimality of two heuristic algorithms based on the Shortest Processing Time among Available job (SPTA) rule, namely, the SPTA-F and SPTA-A rules. In this paper, we try to prove that these rules are also asymptotically optimal for problem F*m|* *no-wait*, *r*_*j*_ *|*Σ*C*_*j*_ in sense of probability limit. An improvement scheme is introduced for the heuristics to enhance the quality of the original solutions. For numerically evaluating the experimental results, new lower bound is provided for the problem. At the end of the paper, computational results demonstrate the convergence of the heuristics and the performance of the improvement scheme.

The remainder of the paper is organized as follows. The formulated expression of the no-wait flow shop is given in Section 2. The asymptotic analysis on the SPTA-based algorithms is introduced in Section 3. The improvement scheme and new lower bound are presented in Section 4. Some computational results are provided in Section 5 and this paper closes with the conclusion in Section 6.

## 2. Problem Statement

Generally, a no-wait flow shop involves *m* machines in series and *n* jobs to be executed. Each job requires to be sequentially processed on each of the *m* machines without preemption. The processing time of job *j*, *j* = 1, 2, …, *n*, on machine *i*, *i* = 1,2,…, *m*, is *p*(*i*, *j*). It is supposed that all the processing times are independently and identically distributed (i.i.d.) random variables defined on interval (0, 1]. The jobs arrive to the system with a release date *r*_*j*_, which is the earliest time when the job is available. A regular assumption on the release dates is that they are nonnegative. The permutation schedule is adopted; that is, the jobs pass the whole system maintaining the sequence that they are processed on the first machine. Let *S*(*i*, *j*) and *C*(*i*, *j*) be the starting time and completion time of job *j* on machine *i*, *i* = 1,2,…, *m*, *j* = 1,2,…, *n*, respectively. There is no intermediate storage between successive machines. The retention time of job *j* on machine *i* is denoted as *q*(*i*, *j*) = *p*(*i*, *j*) + max⁡{0, *C*(*i*, *j*) − *S*(*i*, *j*) − *p*(*i*, *j*)}, where the value of maximum is zero as no blocking occurs. The objective value of algorithm *H* is denoted as *Z*(ALG) and the optimal value is denoted as *Z*(OPT). The objective is to find a schedule that optimizes the total completion time (TCT) for all jobs on the final machine.

## 3. Asymptotic Analysis on the SPTA-Based Algorithms 

In this section, we implement performance analysis on the SPTA-F and SPTA-A rules for the no-wait flow-shop TCT problem. The mathematical proof reveals the asymptotic optimality of these SPTA-based algorithms.

### 3.1. Asymptotic Optimality of the SPTA-F Algorithm

The SPTA-F rule schedules the job with the minimum value of *p*(1, *j*), *j* = 1,2,…, *n*, among the available ones whenever the first machine is idle. Without regard to the extra time (caused by blocking) when jobs remain on the machines, the total time on the first machine can be treated as a lower bound (LB1) for the problem, where the asymptotic optimality of LB1 for the associated single machine problem has been provided by Kaminsky and Simchi-Levi [11]:

(1) $$\begin{array}{c} Z\left(\text{LB}1\right)=\sum_{j=1}^{n}\underset{1\leq x\leq j}{\max}\left\{r_{x}+\sum_{k=x}^{j}p\left(1,k\right)\right\}, \end{array}$$

where *Z*(LB1) denotes the objective value of LB1. Therefore, the following results are presented.

**Theorem 1**

Let the processing times of job *j*, *p*(*i*, *j*), *j* = 1,2,…, *n*, *i* = 1,2,…, *m*, be independent random variables having the same continuous distribution defined on (0, 1]. With probability one, one has

(2) $$\begin{array}{c} \underset{n\to \infty }{\lim}\frac{Z\left(LB1\right)}{n^{2}}=\underset{n\to \infty }{\lim}\frac{Z\left(OPT\right)}{n^{2}}=\underset{n\to \infty }{\lim}\frac{Z\left(SPTA\text{-}F\right)}{n^{2}}, \end{array}$$

where *Z*(SPTA-F) is the objective value obtained by the SPTA-F rule.

**Proof**

Denote the completion time of job *j*, *j* = 1, 2, …, *n*, on the first machine in LB1 as *C*_*j*_(LB1). Therefore, we have

(3) C(m,j)−Cj(LB1)=max⁡1≤x1≤l1≤⋯≤lm≤j{rx1+∑k=x1l1q(1,k)+∑k=l1l2q(2,k)+⋯          +∑k=lmjq(m,k)}−max⁡1≤x≤j{rx2+∑k=x2jp(1,k)}≤max⁡1≤x1≤l1≤⋯≤lm−1≤j{(∑k=x1l1q(1,k)+∑k=l1l2q(2,k)+⋯            +∑k=lm−1jq(m,k))−∑k=x1jp(1,k)}≤max⁡1≤x1≤l2≤⋯≤lm−1≤j{∑k=x1l1(q(1,k)−p(1,k))           +∑k=l1l2(q(2,k)−p(1,k))+⋯           +∑k=lm−1j(q(m,k)−p(1,k))}+(m−1).

Note that

(4) q(i,j)=p(i,j)+max⁡{0,C(i,j)−S(i,j)−p(i,j)}≤p(i+1,j−1).

Under the assumption of Theorem 1, for every *j*, *j* = 1,2,…, *n*, with probability one, Xia et al. [12] proved that

(5) $$\begin{array}{c} \underset{n\to \infty }{\lim}\frac{\text{ma}{\text{x}}_{1\leq l_{1}\leq l_{2}\leq n}\left|\sum_{k=l_{1}}^{l_{2}}\left(p\left(a,k\right)-p\left(1,k\right)\right)\right|}{n}=0, \end{array}$$

where *a* = 2,3,…, *m*. With inequalities (4) and (5), dividing *n* on both sizes of (3) and taking limit, we have

(6) $$\begin{array}{c} 0\leq \underset{n\to \infty }{\lim}\frac{C\left(m,j\right)-C_{1}\left(1,j\right)}{n}=0. \end{array}$$

For the objective values, we have

(7) Z(SPTA-F)−Z(OPT)≤Z(SPTA-F)−Z(LB1)≤∑j=1n(C(m,j)−Cj(LB1))≤nmax⁡1≤j≤n{C(m,j)−Cj(LB1)}.

Noting limit (6), dividing *n* ^2^ on the both sides of (7), and taking limit, we can get the result.

### 3.2. Asymptotic Optimality of the SPTA-A Algorithm

The SPTA-A rule schedules the job with the minimum value of *P*_*j*_ = ∑_*i*=1_^*m*^ *p*(*i*, *j*), *j* = 1,2,…, *n*, among the available ones whenever the first machine is idle. Similarly, the associated lower bound (LB2) is provided for the problem:

(8) $$\begin{array}{c} Z\left(\text{LB}2\right)=\sum_{j=1}^{n}\underset{1\leq x\leq j}{\max}\left\{r_{x}+\frac{1}{m}\sum_{k=x}^{j}\sum_{i=1}^{m}p\left(i,k\right)\right\}, \end{array}$$

where *Z*(LB2) denotes the objective value of LB2. For LB1 and LB2, Xia et al. [12] presented the following result.

**Lemma 2**

Under the assumption of Theorem 1, for a given instance of the flow shop problem and its corresponding optimal solutions of LB1 and LB2, with probability one, one has

(9) $$\begin{array}{c} \underset{n\to \infty }{\lim}\frac{Z^{*}\left(L1\right)}{n^{2}}=\underset{n\to \infty }{\lim}\frac{Z^{*}\left(LB2\right)}{n^{2}}, \end{array}$$

where Z*(LB1) and Z*(LB2) denote the optimal values of LB1 and LB2, respectively.

With this lemma, one can obtain the asymptotic optimality for the SPTA-A rule.

**Theorem 3**

Under the assumption of Theorem 1, with probability one, one has

(10) $$\begin{array}{c} \underset{n\to \infty }{\lim}\frac{Z\left(LB2\right)}{n^{2}}=\underset{n\to \infty }{\lim}\frac{Z\left(OPT\right)}{n^{2}}=\underset{n\to \infty }{\lim}\frac{Z\left(SPTA\text{-}F\right)}{n^{2}}, \end{array}$$

where *Z*(SPTA-A) is the objective value obtained by the SPTA-A rule.

**Proof**

For a given problem instance, we have

(11) Z(SPTA-A)−Z(OPT)≤Z(SPTA-A)−Z(LB2)=(Z(SPTA-A)−Z(LB1)) +(Z(LB2)−Z(LB1)).

Referring to the proof of Theorem 1, we obtain

(12) $$\begin{array}{c} \underset{n\to \infty }{\lim}\frac{Z\left(\text{LB}1\right)}{n^{2}}=\underset{n\to \infty }{\lim}\frac{Z\left(\text{SPTA-A}\right)}{n^{2}}. \end{array}$$

Noting Lemma 2 and limit (12), dividing *n* ^2^ on the both sides of (7), and taking limit, we can get the result.

## 4. Improvement Scheme and Lower Bound

This section provides an improvement based on the properties of job sequencing to enhance the performance of the SPTA-based algorithms. For numerical verifying the asymptotic optimality of the algorithms, a new lower bound is presented.

### 4.1. Improvement Scheme for SPTA-Based Algorithm

Before giving the improvement scheme, several properties for problem F2*|no-wait*, *r*_*j*_ *|*Σ*C*_*j*_ are introduced first.

**Property 1**

For F2*|no-wait*, *r*_*j*_ *|*Σ*C*_*j*_, if jobs *a* and *b* satisfy (1)  *r*_*a*_ ≤ *r*_*b*_, (2)  *p*(1, *a*) ≤ *p*(2, *b*) and *p*(1, *b*) ≤ *p*(2, *a*), and (3)  *p*(1, *a*) − *p*(1, *b*)+(*p*(2, *a*) − *p*(2, *b*))/2 ≥ *r*_*a*_ − *r*_*b*_, then the optimal sequence is that job *a* is scheduled before job *b*.

**Proof**

Suppose the optimal sequence is that job *b* is scheduled before job *a*. The completion times of job *j*  (*j* = *a*, *b*) in sequences {*a*, *b*} and {*b*, *a*} are *C*_*j*_(1) and *C*_*j*_(2), respectively. For sequence {*a*, *b*}, we have

(13) $$\begin{array}{c} C_{a}\left(1\right)+C_{b}\left(1\right)=2r_{a}+2p\left(1,a\right)+2p\left(2,a\right)+p\left(2,b\right). \end{array}$$

For sequence {*b*, *a*}, we have

(14) $$\begin{array}{c} C_{a}\left(2\right)+C_{b}\left(2\right)=2r_{b}+2p\left(1,b\right)+2p\left(2,b\right)+p\left(2,a\right). \end{array}$$

Because sequence {*b*, *a*} is optimal,

(15) $$\begin{array}{c} C_{a}\left(1\right)+C_{b}\left(1\right)>{\text{C}}_{a}\left(2\right)+{\text{C}}_{b}\left(2\right). \end{array}$$

Hence,

(16) $$\begin{array}{c} p\left(1,a\right)-p\left(1,b\right)+\frac{p\left(2,a\right)-p\left(2,b\right)}{2}<r_{a}-r_{b}, \end{array}$$

which leads to a contradiction.

**Property 2**

For F2*|no-wait*, *r*_*j*_ *|*Σ*C*_*j*_, if jobs *a* and *b* satisfy (1)   *r*_*a*_ ≤ *r*_*b*_, (2)  *p*(2, *a*) ≤ *p*(1, *b*) and *p*(1, *a*) ≤ *p*(2, *b*), and (3)  *p*(1, *a*)−(*p*(1, *b*) + *p*(2, *b*))/2 ≥ *r*_*a*_ − *r*_*b*_, then the optimal sequence is that job *a* is scheduled before job *b*.

**Proof**

Suppose the optimal sequence is that job *b* is scheduled before job *a*. The completion times of job *j*  (*j* = *a*, *b*) in sequences {*a*, *b*} and {*b*, *a*} are *C*_*j*_(1) and *C*_*j*_(2), respectively. For sequence {*a*, *b*}, we have

(17) Ca(1)+Cb(1) =2ra+2p(1,a)+p(2,a)+p(1,b)+p(2,b).

For sequence {*b*, *a*}, we have

(18) $$\begin{array}{c} C_{a}\left(2\right)+C_{b}\left(2\right)=2r_{b}+2p\left(1,b\right)+2p\left(2,b\right)+p\left(2,a\right). \end{array}$$

Because sequence {*b*, *a*} is optimal,

(19) $$\begin{array}{c} C_{a}\left(1\right)+C_{b}\left(1\right)>C_{a}\left(2\right)+C_{b}\left(2\right). \end{array}$$

Hence,

(20) $$\begin{array}{c} p\left(1,a\right)-\frac{p\left(1,b\right)+p\left(2,b\right)}{2}<r_{a}-r_{b}, \end{array}$$

which leads to a contradiction.

**Property 3**

For F2*|no-wait*, *r*_*j*_ *|*Σ*C*_*j*_, if jobs *a* and *b* satisfy (1)  *r*_*a*_ ≤ *r*_*b*_, (2)  *p*(1, *b*) ≤ *p*(2, *a*) and *p*(2, *b*) ≤ *p*(1, *a*), and (3)  (*p*(1, *a*) − *p*(1, *b*))/2 + (*p*(2, *a*) − *p*(1, *b*))/2 ≥ *r*_*a*_ − *r*_*b*_, then the optimal sequence is that job *a* is scheduled before job *b*.

**Proof**

Suppose the optimal sequence is that job *b* is scheduled before job *a*. The completion times of job *j*  (*j* = *a*, *b*) in sequences {*a*, *b*} and {*b*, *a*} are *C*_*j*_(1) and *C*_*j*_(2), respectively. For sequence {*a*, *b*}, we have

(21) $$\begin{array}{c} C_{a}\left(1\right)+C_{b}\left(1\right)=2r_{a}+2p\left(1,a\right)+2p\left(2,a\right)+p\left(2,b\right). \end{array}$$

For sequence {*b*, *a*}, we have

(22) Ca(2)+Cb(2) =2rb+2p(1,b)+p(2,b)+p(1,a)+p(2,a).

Because sequence {*b*, *a*} is optimal,

(23) $$\begin{array}{c} C_{a}\left(1\right)+C_{b}\left(1\right)>C_{a}\left(2\right)+C_{b}\left(2\right). \end{array}$$

Hence,

(24) $$\begin{array}{c} \frac{p\left(1,a\right)-p\left(1,b\right)}{2}+\frac{p\left(2,a\right)-p\left(1,b\right)}{2}<r_{a}-r_{b}, \end{array}$$

which leads to a contradiction.

**Property 4**

For F2*|no-wait*, *r*_*j*_ *|*Σ*C*_*j*_, if jobs *a* and *b* satisfy (1)  *r*_*a*_ ≤ *r*_*b*_, (2)  *p*(2, *a*) ≤ *p*(1, *b*) and *p*(2, *b*) ≤ *p*(1, *a*), and (3)  (*p*(1, *a*) − *p*(1, *b*))/2 ≥ *r*_*a*_ − *r*_*b*_, then the optimal sequence is that job *a* is scheduled before job *b*.

**Proof**

Suppose the optimal sequence is that job *b* is scheduled before job *a*. The completion times of job *j*  (*j* = *a*, *b*) in sequences {*a*, *b*} and {*b*, *a*} are *C*_*j*_(1) and *C*_*j*_(2), respectively. For sequence {*a*, *b*}, we have

(25) Ca(1)+Cb(1) =2ra+2p(1,a)+p(2,a)+p(1,b)+p(2,b).

For sequence {*b*, *a*}, we have

(26) $$\begin{array}{c} C_{a}\left(2\right)C_{b}\left(2\right)=2r_{b}+2p\left(1,b\right)+p\left(2,b\right)+p\left(1,a\right)+p\left(2,a\right). \end{array}$$

Because sequence {*b*, *a*} is optimal,

(27) $$\begin{array}{c} C_{a}\left(1\right)+C_{b}\left(1\right)>C_{a}\left(2\right)+C_{b}\left(2\right). \end{array}$$

Hence,

(28) $$\begin{array}{c} \frac{p\left(1,a\right)-p\left(1,b\right)}{2}<r_{a}-r_{b}, \end{array}$$

which leads to a contradiction.

On the basis of these properties, we design an improvement scheme to promote the solution obtained by SPTA-based rules.

The *m* machines are divided into *m* − 1 groups and denote group *g* = {*i* − 1, *i*}, *i* = 1,2,…, *m*. The processing time of job *j* on machine *i*, *i* = 1,2, in group *g* is defined by *p*_*g*_(*i*, *j*). Therefore, the improvement scheme is stated formally as follows.

*Improvement Scheme*

*Step 1.* Generate the initial sequence *π*_0_ with SPTA-based rule and calculate the objective value *Z*_0_. Reindex the jobs from 1 to *n*. 

*Step 2.* Divide the *m* machines into *m* − 1 groups. For each machine group *g* = {*i* − 1, *i*}, *i* = 1,2,…, *m*, execute the following adjustment substeps for sequence *π*_0_. 

*Step 2.1.* For two jobs *a* and *b* with *r*_*a*_ ≤ *r*_*b*_, if one of the following conditions is satisfied: (1) *p*_*g*_(1, *a*) − *p*_*g*_(1, *b*)+(*p*_*g*_(2, *a*) − *p*_*g*_(2, *b*))/2 ≥ *r*_*a*_ − *r*_*b*_, (2)  *p*_*g*_(1, *a*)−(*p*_*g*_(1, *b*) + *p*_*g*_(2, *b*))/2 ≥ *r*_*a*_ − *r*_*b*_, (3)   (*p*_*g*_(1, *a*) − *p*_*g*_(1, *b*))/2 + (*p*_*g*_(2, *a*) − *p*_*g*_(1, *b*))/2 ≥ *r*_*a*_ − *r*_*b*_, and (4)  (*p*_*g*_(1, *a*) − *p*_*g*_(1, *b*))/2 ≥ *r*_*a*_ − *r*_*b*_, then interchange jobs *a* and *b* and calculate the objective value. 

*Step 2.2.* If the objective value obtained in the previous substep is smaller than *Z*_0_, retain the current sequence; otherwise, hold the original sequence unchangeably. 

*Step 3.* Stop the proceeding until all the jobs in each machine group are checked, and select the sequence with the minimum objective value as the final solution.

Obviously, without regard to the no-wait constraint, the improvement scheme can also solve the F*m|* *r*_*j*_ *|*Σ*C*_*j*_ problem.

### 4.2. New Lower Bound

For the F*m|* *r*_*j*_ *|*Σ*C*_*j*_ problem, Bai and Ren [13] presented an asymptotically optimal lower bound, LB-BR, as a substitute for the optimal schedule:

(29) Z(LB-BR)=max⁡{X1,X2,X3},X1=∑j=1nmax⁡1≤x≤j{rx+∑k=xjp(1,k)+∑i=2mp(i,j)},X2=∑j=1nmax⁡1≤x≤j{rx+∑i=1m−1p(i,x)+∑k=xjp(m,k)},X3∑j=1nmax⁡1≤x≤j{rx+1m(∑i=1m((m−i)p(i,x)+(i−1)p(i,j)+∑k=xjp(i,k)))},

where *Z*(LB-BR) denotes the objective value obtained by LB-BR. Obviously, this lower bound can solve problem F*m|*no-wait, *r*_*j*_ *|*Σ*C*_*j*_ directly. But we find that LB-BR is not a real lower bound, and sometimes it may be larger than the optimal solution. Consider the following instance.

**Example 4**

A two-machine flow shop scheduling problem involves two jobs (machines *M*1 and *M*2, jobs *J*1 and *J*2) without release date. The processing times of the jobs are listed as follows:

(30) J1J2M11εM2ε1,

where *ε* is an arbitrary small positive number. The LB-BR in the SPTA-A sequence {*J*1, *J*2} has the objective value of *Z*(LB-BR) = 3 + 2*ε*, but the optimal sequence {*J*2, *J*1} has the optimal solution of *Z*(OPT) = 2 + 3*ε*. Clearly, *Z*(LB-BR) > *Z*(OPT). To avoid such cases, a new lower bound, LB*, is introduced to deal with the flow shop total completion time problem, including no-wait constraint:

(31) $$\begin{array}{c} Z\left(\text{L}{\text{B}}^{*}\right)=\max\left\{Z\left(\text{LB}3\right),Z\left(\text{LB}4\right)\right\}, \end{array}$$

where

(32) $$\begin{array}{c} Z\left(\text{LB}3\right)=\sum_{j=1}^{n}\underset{1\leq x\leq j}{\max}\left\{r_{x}+\sum_{k=x}^{j}p\left(1,k\right)+\underset{x\leq k\leq j}{\min}\sum_{i=2}^{m}p\left(i,k\right)\right\}, \end{array}$$

(33) Z(LB4)=∑j=1nmax⁡1≤x≤j{rx+1m(∑i=1m(min⁡x≤k≤j{(m−i)p(i,k)+(i−1)p(i,k)}+∑k=xjp(i,k)))}.

Calculating the value of LB* for sequence {*J*1, *J*2}, we have

(34) $$\begin{array}{c} Z\left({\text{LB}}^{*}\right)=2+3\varepsilon =Z\left(\text{OPT}\right). \end{array}$$

## 5. Numerical Simulation

This section designed a series of simulation experiments to show the convergence of the SPTA-based rules and the effectiveness of the improvement scheme for different size problems. In the computational testing, the following parameters are varied:

1. machines: 3, 5, and 10 machines for testing the SPTA-based rules; 10, 15, and 20 machines for testing improvement scheme;
2. jobs: 100, 500, 1000, and 1500 jobs for testing the SPTA-based rules; 10, 20, 50, 80, and 100 jobs for testing improvement scheme;
3. processing times: randomly generated from a discrete uniform distribution on [12, 13] and a discrete normal distribution with expectation 5 and variance 25;
4. release dates: *r*_1_ = 0, *r*_*j*_ + 1 = *r*_*j*_ + *y*, *j* = 2,…, *n*, where *y* is a random variable generated independently from a discrete uniform distribution on [12, 13].

Ten different random trials are performed for each combination of these parameters, respectively. The averages are reported in the tables.

### 5.1. Convergence Trend of SPTA-Based Algorithms

Tables 1 and 2 listed the mean relative ratio (*Z*(SPTA-X) − *Z*(LB*))/*Z*(LB*) for the SPTA-F/A rule, where *Z*(SPTA-X) denotes the SPTA-F and SPTA-A rules. Tables 3 and 4 report the ratio *Z*(LB4)/*Z*(LB3) for the associated lower bound obtained by the SPTA-F/A rule. The data in Tables 1 and 2 showed that the ratios approach zero as the number of jobs increases, indicating that convergence trend is independent of processing time distributions and suggesting that the SPTA-based algorithms are asymptotically optimal. For five machines and uniform distribution, for example, the ratios of the SPTA-F rule dropped from 0.05581 to 0.00895, when the number of jobs increases from 100 to 1500. The ratios in Tables 3 and 4 revealed that one lower bound dominating the other is impossible. With Lemma 2, we can conclude that the two lower bounds are asymptotically equal when the job number is sufficiently large for the same instances.

### 5.2. Performance of Improvement Scheme

As the experiments in Section 5.1 demonstrate nonsignificant difference for the SPTA-F and SPTA-A rules, we select one of them for testing the performance of the improvement scheme.  Table 5 listed the mean relative ratio (*Z*(SPTA-F) − *Z*(IS))/*Z*(IS) for the improvement scheme, where *Z*(IS) denotes the objective value obtained by the improvement scheme. The data in Table 5 indicate that the improvement scheme enhances the performance of the SPTA-F rule effectively for moderate-sized problems. As the problem scale continues to enlarge, the improvement is weakened and the running time lengthens. Therefore, obtaining the near-optimal solution with the SPTA-based rules directly for large-scale problems is more practical.

## 6. Conclusions

In this paper, we first proved that the SPTA-based algorithms are asymptotically optimal as the problem size tends to infinity for problem F*m|* *no-wait*, *r*_*j*_ *|*Σ*C*_*j*_. And then, an improvement scheme based on job swap is presented to boost the performance of the SPTA-based algorithms for moderate scale problems. To evaluate the convergence trend of the algorithms, new lower bound is provided. Computational results show the effectiveness of the proposed algorithms

## Figures and Tables

**Table 1:** Convergence results of the SPTA-F rule.

	Uniform			Normal		
	*m* = 3	*m* = 5	*m* = 10	*m* = 3	*m* = 5	*m* = 10
*n* = 100	0.03256	0.05581	0.08688	0.05174	0.08921	0.12010
*n* = 500	0.00857	0.01715	0.03040	0.01257	0.01354	0.07037
*n* = 1000	0.00537	0.01095	0.02180	0.00821	0.01017	0.04227
*n* = 1500	0.00434	0.00895	0.01904	0.00659	0.00973	0.03215

**Table 2:** Convergence results of the SPTA-A rule.

	Uniform			Normal		
	*m* = 3	*m* = 5	*m* = 10	*m* = 3	*m* = 5	*m* = 10
*n* = 100	0.03450	0.05207	0.09267	0.05764	0.07912	0.11909
*n* = 500	0.01450	0.02920	0.05914	0.02106	0.03173	0.10651
*n* = 1000	0.01240	0.02481	0.04617	0.01717	0.02673	0.10510
*n* = 1500	0.01154	1.02352	0.04005	0.01513	0.02479	0.10427

**Table 3:** Z(LB4)/Z(LB3) in the SPTA-F sequence.

	Uniform			Normal		
	*m* = 3	*m* = 5	*m* = 10	*m* = 3	*m* = 5	*m* = 10
*n* = 100	0.98993	0.98644	0.97953	0.98796	0.98837	0.99573
*n* = 500	0.98294	0.974864	0.96953	0.98465	0.98724	0.99298
*n* = 1000	0.98162	0.97380	0.96800	0.98317	0.98721	0.98973
*n* = 1500	0.98147	0.97206	0.96676	0.98307	0.98371	0.98732

**Table 4:** Z(LB4)/Z(LB3) in the SPTA-A sequence.

	Uniform			Normal		
	*m* = 3	*m* = 5	*m* = 10	*m* = 3	*m* = 5	*m* = 10
*n* = 100	1.00079	1.00556	1.00120	1.00053	1.00215	1.00207
*n* = 500	1.00062	1.00085	1.00360	1.00021	1.00059	1.00107
*n* = 1000	1.00006	1.00104	1.00127	1.00011	0.00073	1.00111
*n* = 1500	1.00008	1.00016	1.00028	1.00007	1.00017	1.00033

**Table 5:** Results of Z(SPTA-F)/Z(IS).

	Uniform			Normal		
	*m* = 10	*m* = 15	*m* = 20	*m* = 10	*m* = 15	*m* = 20
*n* = 10	0.08256	0.08437	0.09334	0.03769	0.03886	0.04416
*n* = 20	0.04942	0.05705	0.05711	0.03697	0.03873	0.04422
*n* = 50	0.03833	0.04024	0.04384	0.03601	0.03826	0.04375
*n* = 80	0.03309	0.03403	0.03431	0.03599	0.03811	0.03970
*n* = 100	0.02817	0.02861	0.03009	0.03500	0.03810	0.03843
